# Inhibitory Effect of Baicalin on iNOS and NO Expression in Intestinal Mucosa of Rats with Acute Endotoxemia

**DOI:** 10.1371/journal.pone.0080997

**Published:** 2013-12-02

**Authors:** Aiwen Feng, Guangrong Zhou, Xiaoming Yuan, Xinli Huang, Zhengyuan Zhang, Ti Zhang

**Affiliations:** 1 Department of Gastrointestinal Surgery, Huai'an First People’s Hospital, Nanjing Medical, University, Huai'an, P. R. China; 2 Department of General Surgery, the First Affiliated Hospital with Nanjing Medical University, Nanjing, P. R. China; Ohio State University, United States of America

## Abstract

The mechanism by which baicalin modulated the expression of inducible nitric oxide synthase (iNOS) and nitric oxide (NO) in the mucosa of distal ileum was investigated in a rat model of acute endo-toxemia induced by intraperitoneal injection of bacterial lipopolysaccharide (LPS). The experiment demonstrated that LPS upregulated iNOS mRNA and protein expression as well as NO produc-tion (measured as the stable degradation production, nitrites). LPS not only increased toll-like receptor 4 (TLR4) and peroxisome proliferator-activated receptor gamma (PPARγ) content, but also activated p38 and activating transcription factor 2 (ATF2) and inactivated PPARγ *via* phosphorylation. Inhibition of p38 signalling pathway by chemical inhibitor SB202190 and small interfering RNA (siRNA) ameliorated LPS-induced iNOS generation, while suppression of PPARγ pathway by SR-202 boosted LPS-elicited iNOS expression. Baicalin treatment (I) attenuated LPS-induced iNOS mRNA and protein as well as nitrites generation, and (II) ameliorated LPS-elicited TLR4 and PPARγ production, and (III) inhibited p38/ATF2 phosphorylation leading to suppression of p38 signalling, and (IV) prevented PPARγ from phosphorylation contributing to maintainence of PPARγ bioactivity. However, SR-202 co-treatment (I) partially abrogated the inhibitory effect of baicalin on iNOS mRNA expression, and (II) partially reversed baicalin-inhibited p38 phosphorylation. In summary, baicalin could ameliorate LPS-induced iNOS and NO overproduction in mucosa of rat terminal ileum *via* inhibition of p38 signalling cascade and activation of PPARγ pathway. There existed a interplay between the two signalling pathways.

## Introduction

The mucosa along the intestinal tract, especially the ileum, is susceptible to harmful factors such as LPS (also called endotoxin), which is a major cell wall component of Gram-negative bacteria [1]. LPS and derived pro-inflammatory molecules such asiNOS and NO may impair intestinal mucosa [2], [3]. Severe devastation of intestinal mucosa plays an important role in intestinal barrier dysfunction, bacterial translocation, systemic inflammatory response syndrome and multiple organ dysfunction syndrome [4]. In the whole intestine, iNOS is found to be predominantly expressed in intestinal epithelial cell, then in macrophage, fibroblast, and smooth muscle cell [1], [5].

p38, a member of MAPK superfamily, can trigger inflammatory response when it is activated. p38 and downstream molecules (e.g., ATF2) are actively involved in the formation of intestinal inflam-mation such as colitis and necrotizing enteritis. p38 plays a required role in the induction of iNOS expression in intestinal epithelium [2]. p38 also partially mediates iNOS production in macrophage and fibroblast [6], [7], [8]. In contrast to p38, PPARγ, a member of nuclear hormone receptor superfamily, can suppress inflammation in the context of endogenous and exogenous ligands [9]. In intestinal tract, PPARγ is expressed in intestinal epithelium and macrophage. PPARγ plays an essential role in the inhibition of intestinal inflammation [10].

**Figure 1:**
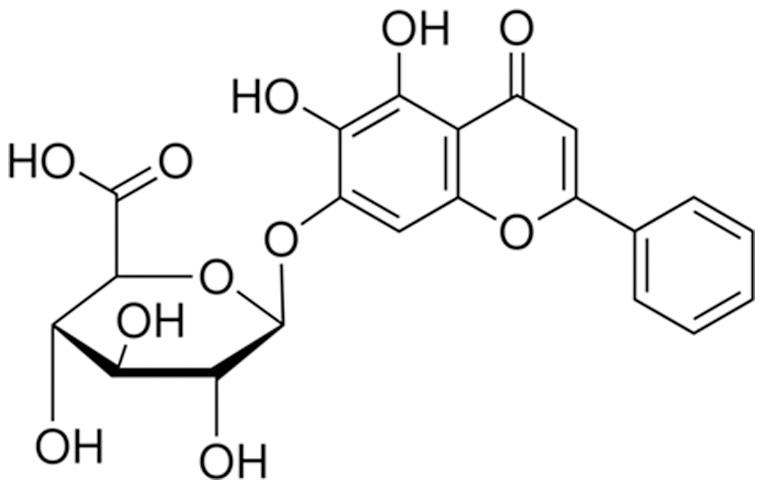
The molecular structure of baicalin.

Baicalin (Fig. 1) is used as a traditional herbal medicine. This flavonoid is purified from the root of plant *Scutellaria baicalensis Georgi.* In vitro, baicalin possesses the antioxidant, antiinflammation, antivirus, antibacteria properties. In vivo, baicalin ameliorates experimental chemical colitis [11], [12], [13] and small intestinal injury in severe acute pancreatitis [14], [15]. Baicalin has been reported to repress p38 phosphorylation [16], [17] and activate PPARγ [18], [19] to attenuate inflammation. Therefore, in this experiment we explored whether baicalin would regulate iNOS and NO expression in small intestinal mucosa *via* modulation of p38 and/or PPARγ pathways in a rat model of acute endotoxemia.

## Materials and Methods

### Animals and experimental design

Seven to eight weeks old male Sprague-Dawley rats (250–300 g) were randomly allocated to six groups (Table 1). Group I was designed as control group and Group II ∼ VI as experimental groups. Group IV and V contained three and two subgroups, respectively. There were seven rats in every group or subgroup. The rats were housed at an ambient temperature of 22–23°C and a humidity of 50%–60%, maintained in a 12h/12h light-dark cycle, and fed with water and standard rodent chow for one week. All animals were fasted overnight before this experimentation. The Animal Care and Use Committee of Nanjing Medical University approved this project.

**Table 1:** Experimental design in this study.

Group	Treatment
I	saline
II	LPS
III	SB202190 plus LPS
IV (three subgroups)	
Subgroup 1	negative control siRNA plus LPS
Subgroup 2	p38 siRNA plus LPS
Subgroup 3	ATF2 siRNA plus LPS
V (two subgroups)	
Subgroup 1	baicalin (25 mg/kg/d) plus LPS
Subgroup 2	baicalin (50 mg/kg/d) plus LPS
VI	baicalin (50 mg/kg/d) plus LPS plus SR-202

### Pretreatment of baicalin, SB202190 and SR-202

SB202190 (a p38 inhibitor, purity ≥ 98%), SR-202 (a PPARγ antagonist, purity ≥ 98%) and baicalin (purity ≥ 97%) purchased from Sigma-Aldrich Co. (St Louis, USA) were used in this study. The three reagents, dissolved in 0.1% v/v DMSO vehicle according to the vendor's guideline, were administered at a single injection per day for consecutive 3 days according to literatures as follows: SB202190 and SR-202 at the same dosage of 3 mg/kg/d, i.p [20] and baicalin at the dosages of 25 and 50 mg/kg/d, i.p. [21] to observe their regulatory effects on LPS-induced inflammation.

### Pretreatment of siRNAs

The siRNA duplexes (Table 2) were synthesized and chemically modified by a commercial vendor (GenePharma, Shanghai, China). These siRNAs were dissolved in sterile PBS solution to a final concentration of 50 μg/ml. The siRNA was delivered by systemic i.p. administration at a dosage of 120 μg/kg/day for consecutive 6 days based on previous literatures [22], [23]. The rats treated with normal saline and negative control siRNA were designed as a control [22]. The functional assessment of siRNA transfection consists of p-p38, p-ATF2 [2] and iNOS expression.

**Table 2:** siRNA duplexes used in this study

Target gene	Sequences of siRNA duplexes
Negative control	5′-UUCUCCGAACGUGUCACGUTT-3′
	5′-ACGUGACACGUUCGGAGAATT-3′
p38α	5′-GGACCUCCUUAUAGACGAAUU-3′
	5′-UUCGUCUAUAAGGAGGUCCUU-3′
ATF2	5′-CCUUCUGUUGUAGAAACAATT-3′
	5′-UUGUUUCUACAACAGAAGGTT-3′

### Induction of acute endotoxemia in rats

LPS from bacterial *Escherichia coli* (serotype O55:B5) was provided by Sigma-Aldrich Co. (St Louis, USA). This reagent was dissolved in sterile physiological saline to a final concentration of 1 mg/ml. Acute endotoxemia was induced by injection of LPS (3 mg/kg, i.p).

### Preparation of ileal mucosal scrapings

The ileum is the portion of maximal induction of iNOS in the intestinal tract [1]. The rodents were anesthetized at 6 hours after the onset of endotoxemia induction. General anesthesia was induced by i.p. injection of ketamine diluted in 0.9% normal saline at a dosage of 100 mg/kg. The abdomen of each rat was incised and distal segment of ileum (about 10 cm in length) was harvested and then rinsed with saline to remove the feces. The intestinal mucosa was swiftly scraped off with a glass slide after the ileum was scissored longitudinally. Decapitation euthanasia of the laboratory rodents were carried out at the end of the surgical procedure.

### Measurement of NO production as nitrites

To determine NO production, equal weight of mucosal scrapings was homogenized in sterile PBS (1 mL), centrifuged supernatants were collected. Analysis for NO production was adopted by the modified Greiss method [24], [25]. Briefly, 210 ml of homogenate was incubated with nitrate reductase enzyme (10 mU) and β-nicotinamide adenine dinucleotide phosphate (NADPH; 1.25 mg/ml) for 30 min at 37 °C. Then the total nitrite in each sample was determined by addition of 200 mU of L-glutamate dehydrogenase, 100 mM ammonium chloride and freshly prepared 4 mM of α-ketogluterate. The mixture was incubated at 37 °C for 10 min followed by addition of 250 ml of Greiss reagent (Enzo Life Sciences, USA) and incubation for another 5 min at 37°C. Samples were centrifuged, and clear supernatants were collected, then optical density was recorded at 550 nm. The amount of NO produced was determined by calibrating a standard curve using sodium nitrite.

### Reverse transcription polymerase chain reaction (RT-PCR)

Total RNA from fresh intestinal mucosal tissue weighted 100 mg was extracted by TransZol lysis provided by TransGen Biotech (Beijing, China) and centrifugated 12000 rpm, 4°C for 10 min. RT-PCR was performed as follows. First-strand cDNA was synthesized by using Anchored Oligo(dT)_18,_ *EasyScript* RT/RI Enzyme Mix and 2 x ES Reaction Mix (TransGen Biotech, Beijing, China) at 42°C for 60 min, 70°C for 15 min. PCR amplification was performed by the hot starting method using *TransFast Taq*DNA Polymerse (TransGen Biotech, Beijing, China). The forward and reverse primers for iNOS were 5′-CTGCAGGTCTTTGACGCTCGG-3′ and 5′-GTGGAACACAGGGGTGATGCT-3′, respectively. After initial denaturation at 95°C for 5 min, 30 cycles of denaturation at 95°C for 30s, annealing at 55°C for 30s, and elongation at 72°C for 1 min, then 72°C for 10 min were performed using a thermal cycler (Perkin-Elmer Corp.). The forward and reverse for beta-actin were 5′-TTCTACAATGAGCTGCGTGTG-3′ and 5′-CTGGAGATACGGTTGTGTCAC-3′, respectively. The PCR products were separated by electrophoresis on a 1.8% agarose gels containing 0.1% ethidium bromide and were visualized by ultraviolet-induced fluorescence. The intensities of the PCR bands were measured by densitometry using Image-Pro Plus 6.0 (Media Cybernetics, USA).

### Western blotting

Total and nuclear proteins were extracted by using RIPA buffer and nuclear extraction kit (Pierce Co.), respectively, in accordance to the manufacturer's guideline. Tissue lysates were sonicated on ice and centrifuged to sediment the particulate materials. Aliquots of 100 μg of total protein or 10 μg of nuclear extract were electrophoresed on SDS-polyacrylamide gels, then transferred to membranes. After being blocked for 1 hr with milk at room temperature, the blots were incubated with primary polyclonal antibodies (Santa Cruz Biotechnology and Abcam) at different dilutions (from 1∶200 to 1∶500). These primary antibodies include anti-iNOS (sc-651), anti-p-p38 (sc-101758), anti-p-ATF2 (ab131106), anti-PPARγ (sc-7196), anti-p-PPARγ (sc-28001-R) and anti-TLR4 (sc-10741). Appro-priate secondary antibody (goat anti-rabbit IgG-HRP; sc-2004) was applied. Protein bands were visualized using a chemiluminescence substrate according to the manufacturer's instructions. The densitometry analysis of the image was performed by Image-Pro Plus 6.0 (Media Cybernetics, USA).

### Statistical analysis

Statistical analyses were performed with SPSS 13.0 for Windows software (SPSS Inc). All the numerical data were expressed as means ± standard deviation. Statistical comparisons were done by using One-Way ANOVA. After homogeneity test of variances were performed, post hoc comparisons were made using LSD tests with significance levels set at *P*<0.05.

## Results

### LPS induced whereas SB202190 and siRNAs decreased iNOS and NO generation

**Figure 2:**
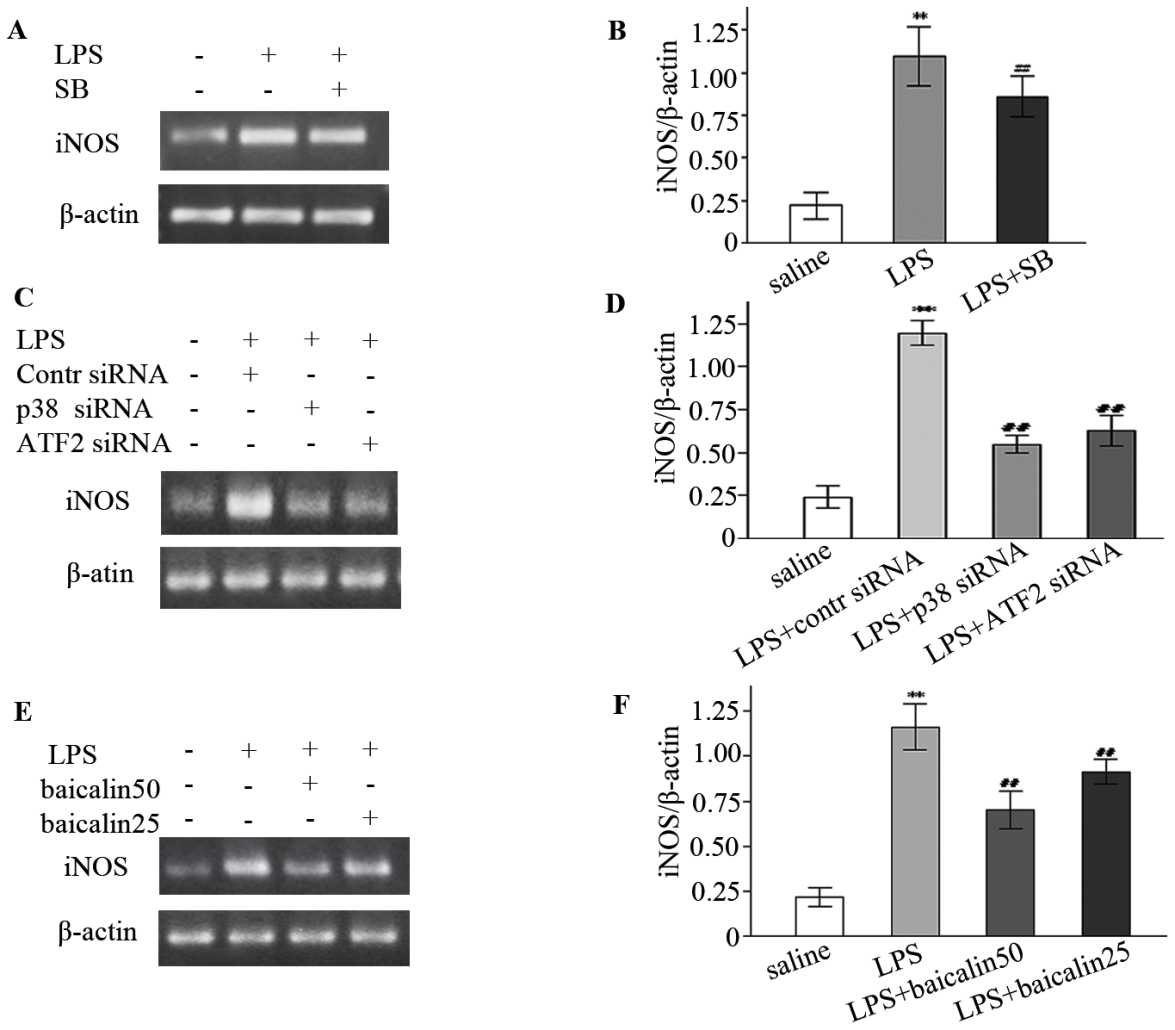
LPS increased but SB202190 (SB), siRNA and baicalin decreased iNOS mRNA expression. A, C, E: Representative gels for iNOS mRNA expression. β-actin is the internal control. B, D, F: Semiquantitation of iNOS mRNA in each group. ^*^ *P*<0.05, ^**^ *P*<0.01 vs. saline group; **^#^** *P*<0.05, **^##^** *P*<0.01 vs. LPS group or LPS plus control (contr) siRNA group.

**Figure 3:**
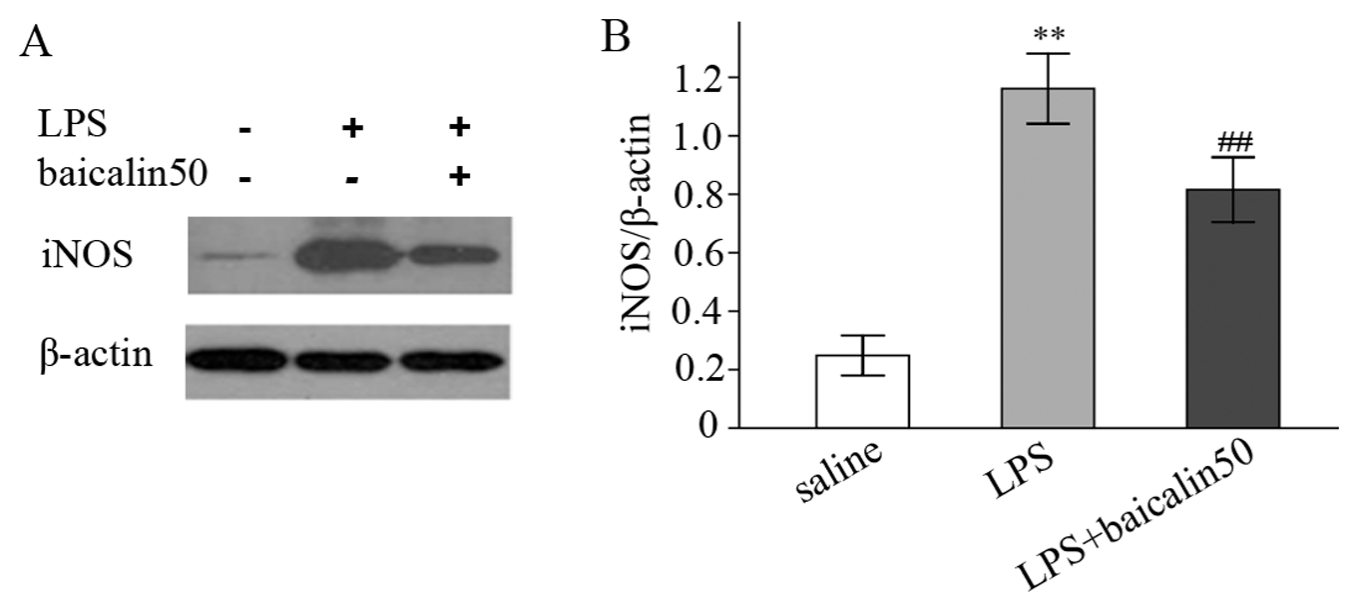
Baicalin inhibited LPS-induced iNOS protein expression. A: Representative gels for iNOS protein expression. B: Semiquantitation of iNOS protein in each group. ^*^ *P*<0.05, ^**^ *P*<0.01 vs. saline group. ^#^ *P*<0.05, ^##^ *P*<0.01 vs. LPS group.

**Figure 4:**
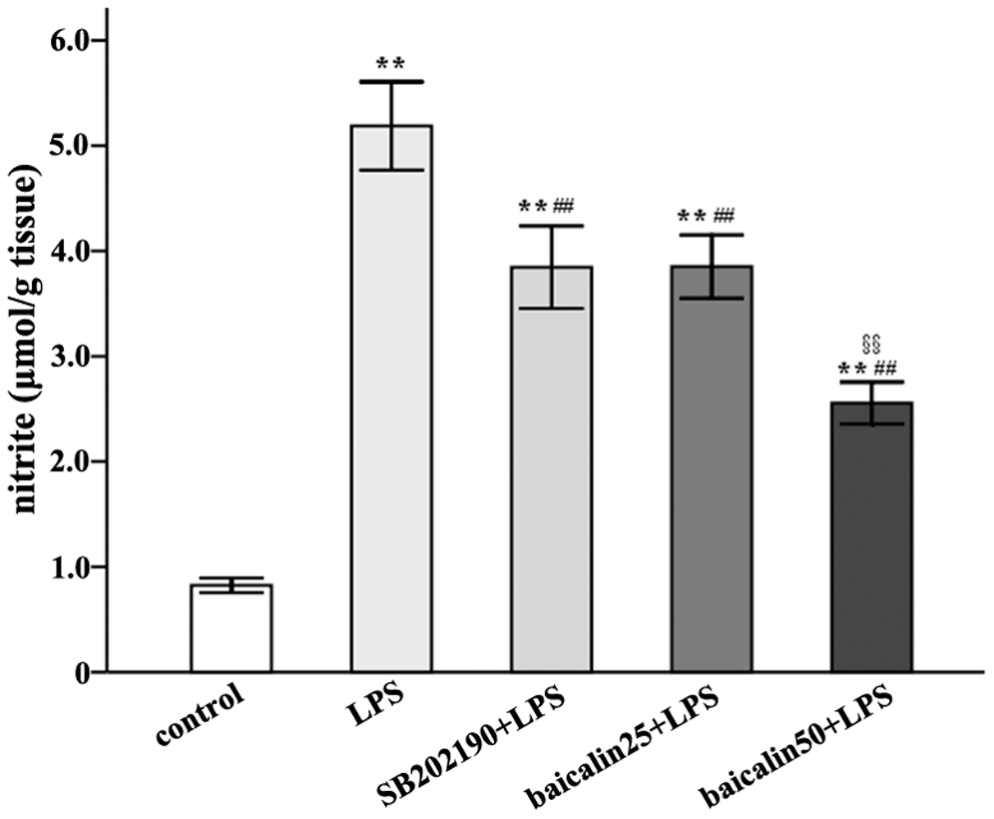
LPS increased but SB202190 and baicalin reduced nitrites production. ^*^ *P*<0.05, ^**^ *P*<0.01 vs. control group; ^#^ *P*<0.05, ^##^ *P*<0.01 vs. LPS; ^§^ *P*<0.05; ^§§^ *P*<0.01 vs. LPS plus baicalin group.

In control subjects, weak expression of iNOS mRNA in ileal mucosa was detected. Rats treated with LPS compared to the control showed higher level of iNOS mRNA (about a 4.0-fold increase) (Fig. 2A/B). Similarly, iNOS protein assay also demonstrated that LPS increased iNOS expression (Fig. 3A/B). In p38 inhibitor studies, SB202190 decreased LPS-elicited iNOS mRNA by 21.6% (Fig. 2A/B). In gene silencing studies, negative control siRNA exert little effect on LPS-induced iNOS mRNA, but siRNAs against p38 and ATF2 genes reduced iNOS mRNA by about 54.3% and 47.6%, respectively (Fig. 2C/D). In nitrites measurement studies, LPS augmented whereas SB202190 reduced nitrites accumulation (Fig. 4).

### LPS elicited whereas siRNA suppressed p38 and ATF2 phosphorylation

**Figure 5:**
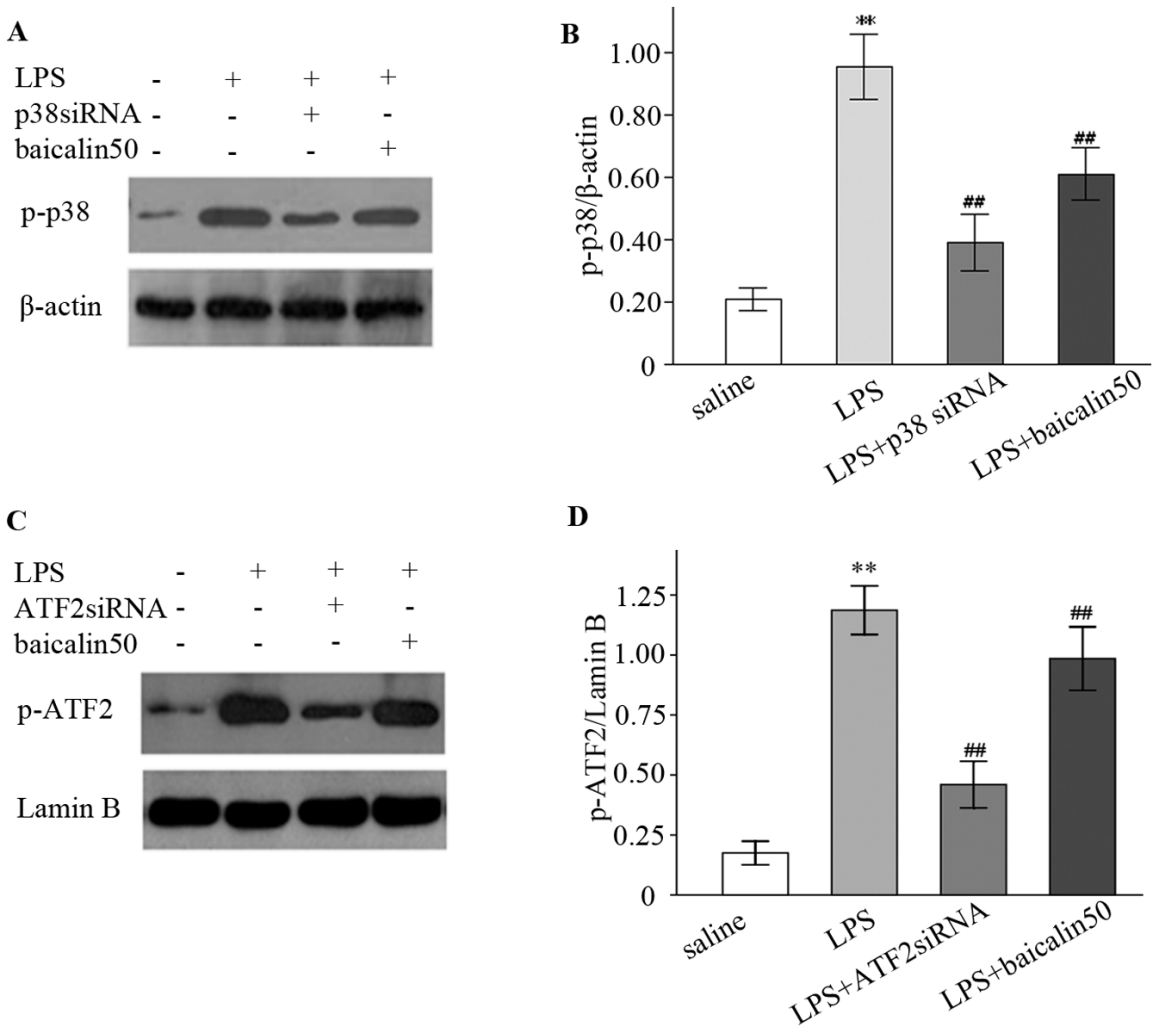
LPS induced but siRNAs and baicalin inhibited p38 and ATF2 phosphorylation. A, C: Representative gels for p-38 and p-ATF2 expression. β-actin and lamin B are the internal controls. B, D: Semiquantitation of p-p38 and p-ATF2 in each group. ^*^ *P*<0.05, ^**^ *P*<0.01 vs. saline group; ^#^ *P*<0.05, ^##^ *P*<0.01 vs. LPS group.

Gene silencing by siRNA has been reported to inhibit p38 and ATF2 synthesis and activation [2], [26], as judged by increase in activating phosphorylation [27]. As shown in Fig. 5A-D, the expression levels of p-p38 and p-ATF2 were low in control subjects but high in endotoxemic rats (a 3.6-fold and 5.7-fold increase, respectively). siRNAs against p38 and ATF2 genes, however, reduced LPS-induced p-p38 and p-ATF2 expression by around 54.3% and 61.2%, respectively.

### Baicalin abrogated LPS-enhanced iNOS and NO production

In Fig. 2E/F, low and high doses of baicalin decreased LPS-induced iNOS mRNA (a 21.5% and 39.8% reduction, respectively). Protein assay revealed that baicalin effectively reduced iNOS protein (Fig. 3A/B). Similarly, nitrites determination also indicated that baicalin decreased LPS-induced nitrites in a dose-dependent manner (Fig. 4).

### Baicalin repressed LPS-mediated p38 and ATF2 phosphorylation

Baicalin can inhibit the activation of some inflammatory molecules [28]. Therefore, the regulatory effect of baicalin on p38/ATF2 signalling cascade was assessed. As shown in Fig. 5A-D, treatment with baicalin repressed LPS-induced phosphorylation of p38 and ATF2 by 36.8% and 25.0%, respectively, indicating baicalin could suppress the activation of p38 pathway.

### Baicalin repressed LPS-induced TLR4 and PPARγ expression

**Figure 6:**
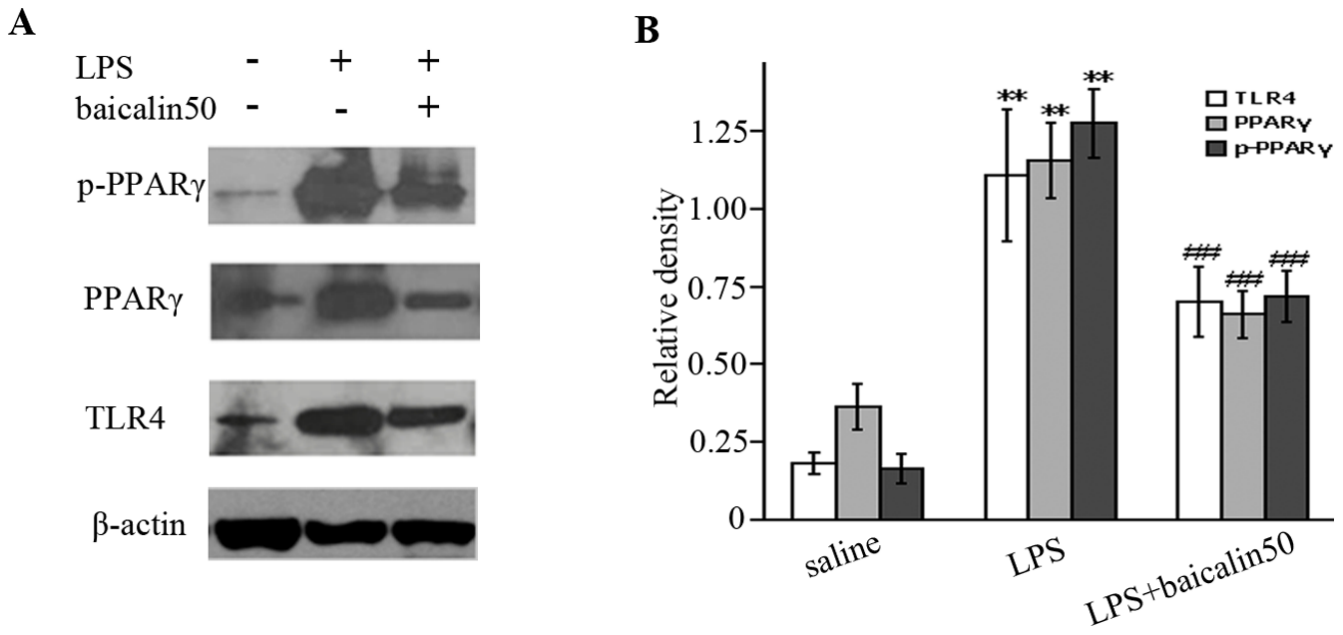
LPS increased but baicalin reduced LPS-mediated TLR4 and PPARγ overexpression and phosphorylation. A: Representative gels for TLR4, PPARγ and p-PPARγ expression. B: Semiquantitation of TLR4, PPARγ and p-PPARγ in each group. ^*^ *P*<0.05, ^**^ *P*<0.01 vs. saline group; ^#^ *P*<0.05, ^##^ *P*<0.01 vs. LPS group.

As demonstrated in Fig. 6A/B, the expression levels of TLR4 and PPARγ protein were low in ileal mucosae of control rats but markedly raised in those of endotoxemic subjects (a 5.0- and 3.2-fold increase, respectively). Baicalin treatment diminished LPS-induced TLR4 and PPARγ production by nearly 36.6% and 40.2%, respectively.

### Baicalin inhibited LPS-induced PPARγ phosphorylation

Phosphorylation of PPARγ would elicit the loss of bioactivity [29], [30], [31]. As illustrated in Fig. 6A/B, the expression level of p-PPARγ was much higher in endotoxemic rats than that in control group. Baicalin treatment partially abrogated the inducible effect of LPS on PPARγ phosphorylation, as indicated by a 45.6% reduction of p-PPARγ production.

### SR-202 had an effect on baicalin-downregulated iNOS and p-p38 expression

**Figure 7:**
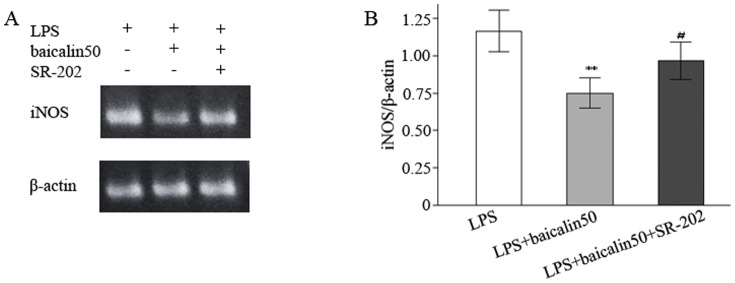
SR-202 antagonised the inhibitory effect of baicalin on iNOS mRNA expression. A: Representative gels for iNOS mRNA expression. B: Semiquantitation of iNOS in each group. ^*^ *P*<0.05, ^**^ *P*<0.01 vs. LPS group. ^#^ *P*<0.05, ^##^ *P*<0.01 vs. LPS plus baicalin group.

**Figure 8:**
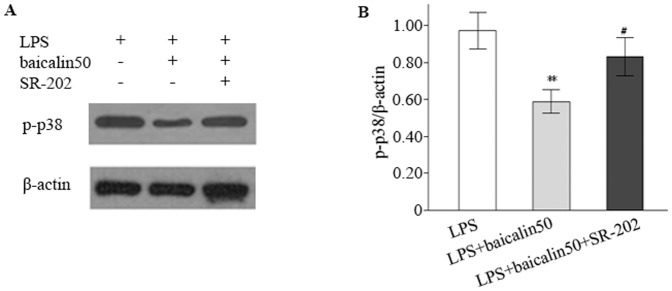
SR-202 antagonised the inhibitory effect of baicalin on p38 phosphorylation. A: Representative gels for p-p38 expression. B: Semiquantitation of p-p38 in each group. ^*^ *P*<0.05, ^**^ *P*<0.01 vs. LPS group; ^#^ *P*<0.05, ^##^ *P*<0.01 vs. LPS plus baicalin group.

SR-202 was used to test the hypothesis that baicalin might suppress iNOS mRNA *via* activation of PPARγ. As illustrated in Fig. 7A/B, the reduced expression of iNOS mRNA by baicalin was partially reversed by SR-202 (an increase of 28.3%). Finally, we investigated the effect of SR-202 on p38 signalling. The results suggested that the decreased expression of p-p38 by baicalin was partially reversed by SR-202 (a increase of 40.3%) (Fig. 8A/B), indicating that SR-202 could increase p-p38 production.

## Discussion

In the intestine, iNOS is a key enzyme for NO production in intestinal mucosal tissue. iNOS is expressed in a small amount and plays a role in intestinal mucosal integrity under physiological conditions [32]. Under pathological conditions, excessive iNOS and NO, however, are harmful to intestinal mucosal barrier [5], [32], [33], [34]. LPS has been confirmed to be a powerful stimulator for iNOS generation [1], [35]. In this rat model, we also demonstrated that LPS upregulated iNOS mRNA and protein as well as NO (measured as nitrites) production, indicating that the intestinal epithelial cell, fibroblast and macrophage may overexpress iNOS and NO in response to endotoxin. Thus, it is of significance to downregulate iNOS production during acute endotoxemia.

Baicalin is a Chinese traditional medicine. Many experiments have suggested that this flavonoid has the property of antiinflammation including ameliorating intestinal injury [11]. In this study, we also demonstrated that pretreatment with baicalin could effectively reduce iNOS and nitrites over-production induced by LPS, suggesting that baicalin colud counteract the noxious effect of LPS on intestinal mucosal barrier to some extend.

LPS binds to receptor TLR4 and then triggers iNOS gene transcription *via* activation of different signalling cascades (e.g., NF-κB, MAPKs). p38 as an important member of MAPK family plays a role in the induction of iNOS gene transcription in the enterocyte and macrophage *in vitro* and *in vivo* [2], [8], [35] through activation of downstream transcriptional factors such as ATF2. Therefore, we aimed to confirm that LPS might elicit iNOS expression in intestinal mucosae of rats *via* activation of p38 signalling pathway. Both p38 inhibitor and gene silencing methods were applied to interefere with p38 signaling pathway. The results demonstrated that LPS induced p38 and ATF2 phosphorylation giving rise to the activation of p38/ATF2 signalling cascade, while pretreatment with SB202190 and siRNAs targeting against p38 and ATF2 genes reduced LPS-induced iNOS overproduction. Nitrites determination also revealed that the upregulatory effect of LPS on nitrites was partially reversed by SB202190. To further assess the effect of baicalin on p38 pathway, we measured the expression levels of p-p38 and p-ATF2 in ileal mucosal scrapings. As expected, baicalin challenge partially counteractd the inducible effect of LPS on p38 and ATF2 phosphorylation. Therefore, baicalin might play as a suppressor of p38 signalling pathway.

Differing from p38, PPARγ possesses the anti-inflammation property. The increase in quantity and activation of bioactivity of PPARγ contribute to attenuating intestinal inflammation and protecting intestinal barrier [36], [37]. LPS not only binds to but also increases receptor TLR4, which is a powerful inducer for PPARγ generation in the intestinal epithelium [38], [39], [40]. We demonstrated that TLR4 and PPARγ were lowly expressed in ileal mucosae of control rats but significantly highly in LPS-treated subjects, which was consistent with other studies [36], [38]. Therefore, upregulation of PPARγ might be a feedback mechanism of self-protection during acute endotoxemia. The phosphorylation of PPARγ, however, may result in the loss of bioactivity [29], [41]. Our rodent model revealed that LPS augmented phosphrylation of PPARγ, indicative of an adverse effect of LPS on PPARγ. Thus, it makes sense to increase and activate PPARγ as well as prevent PPARγ from phosphorylation.

Baicalin has been shown to possess the capacity to decrease or increase PPARγ generation [18], [42]. Our results showed that baicalin did not increase LPS-induced PPARγ; On the contrary, it decreased the generation of PPARγ. In addition, baicalin was found to partially ablolish the inducible effect of LPS on TLR4 production, which was in line with previous literatures [43]. Thus, baicalin's reducing PPARγ was likely associated with its inhibiting LPS-induced TLR4 overexpression.

Of note, baicalin has been reported to efficiently protect intestinal mucosa of rats with severe acute pancretitis [15], [44] through suppression of production of proinflammatory molecules such as LPS, NO and TNFα [15], [45]. Interestingly, this protective action of baicalin is similar to somatostatin and its analogue octretide [15], [44], [46]. Somatostatin and analogues exert inhibitory effect on inflammation [47] and LPS is a stimulator for somatostatin production [48], [49]. In addition, somatostatin may upregulate PPARγ expression [50]. Hence, baicalin's decreasing PPARγ expression also might likely be related to its reprssing LPS and LPS-derived somatostatin production.

In view of baicalin's serving as a role of PPARγ activator [18], [19], We speculated that baicalin might modulate iNOS expression *via* activation of PPARγ pathway. SR-202 was used to test this hypothesis. The result demonstrated that SR-202 treatment partially antagonised the inhibitory effect of baicalin, as indicated by the significant difference of iNOS mRNA expression between Group V (subgroup 2) and Group VI, suggesting that baicalin served as an activator of PPARγ.

Phosphorylation of PPARγ gives rise to the loss of bioactivity. In this experiemen LPS was observed to induce PPARγ phosphorylation, but this adverse effect of LPS was partially repressed by baicalin. Moreover, PPARγ can also be phosphorylated by several kinases (e.g., PKA, MAPKs and AMPK) [51]. Thus, suppressing inactivation of PPARγ by baicalin might be related to counteraction to LPS and inhibition of p38 phosporylation

Finally, we probed if there existed a crosslink between p38 and PPARγ signalling pathways. PPARγ negatively interferes with the activation of proinflammatory signallings (e.g., NF-κB, MAPKs, and STAT-1) [9]. SR-202 was used to antagonize PPARγ pathway. As expected, SR-202 partially reversed baicalin-mediated downexpression of p-p38. Our study showed that p38 induced PPARγ inactivation, whereas PPARγ in turn suppressed p38 activation, indicating of a crosslink between the two signallings.
